# Supplementary material for: The Benefit of Sodium-Glucose Co-Transporter Inhibition in Heart Failure: The Role of the Kidney
Source: Int J Mol Sci. 2022 Oct 9;23(19):11987. doi: 10.3390/ijms231911987 (PMC9569705; doi:10.3390/ijms231911987)
Supplement: Supplementary file 1 [file ijms-23-11987-s001.zip › ijms-1898962-supplementary.pdf]

Supplementary material

Table

The table is a comprehensive collection of the data generated from cardiovascular outcomes trials performed with sodium glucose cotransporter 2 inhibitors in diabetics and not diabetics, bearing different cardiovascular conditions. It includes patient selection criteria, sample size, gender distribution, study duration, study primary and secondary end points. These latter focus on heart failure exacerbation, adverse renal outcomes (detailing criteria for estimation of the variable) and of statistical analysis result on cardiovascular and global mortality. The time of appearance of statistically significant difference in the primary study end-point is reported when available. *High CV (cardiovascular) Risk: established atherosclerotic cardiovascular disease involving the coronary, cerebrovascular, or peripheral arterial systems. **3 points MACE: CV death (D), non-fatal MI or non-fatal stroke; DECLARE TIMI 58 (NCT01730534) >40% decrease in eGFR or<60 ml/min/1.73 m2, dialysis or death from renal or cardiovascular cause. ***Composite renal adverse outcomes: dialysis, transplantation, or a sustained estimated GFR of <15 ml per minute per 1.73 m2, a doubling of the serum creatinine level, or death from renal or cardiovascular causes.

Abbreviations: AF: atrial fibrillation; CKD: chronic kidney disease; CV: cardiovascular; CVD: cardiovascular disease; CVRF: cardiovascular risk factor; Dapa: dapaglifozin; Empa: empaglifozin; EP: end-point; HF: heart failure; eGFR: estimated glomerular filtration rate; HbA1c: glycated hemoglobin; HFh: heart failure hospitalization; LVEF: left ventricular ejection fraction; MACE: major adverse cardiovascular events; NA: not available; NCT: number of clinical trial on https://www.clinicaltrials.gov/; NT-proBNP: amino terminal brain natriuretic peptide; PCB: placebo; T2DM: type2 diabetes mellitus; UACR: urinary albumin/creatinine ratio.

Study (drug)	Study Entry Criteria	Enrolled Subjects (male/female)	Average Age Years	Average Follow up (years)	Primary Endpoint	Primary Endpoint HR (95% CI) p (statistical significance)	CV Death/ Overall Death HR (95% CI)	HF Adverse events: HFh or HF urgent visit	Renal Adverse Outcomes	Mean basal egfr (ml/min/1.73 m^2)	UACR mg/g of creat (IQR).	Time of Statistically Significant Difference Appearance in Study End-Point
EMPA-REG OUTCOME [39] (Empaglifozin)	• T2DM<br>• High CV Risk*<br>• eGFR ≥ 30 (MDRD)	7020 (5016/2004)	63	3.1	3 points MACE**	0.86<br>(0.74, 0.99)<br>0.0382	0.62<br>0.49-0.77<br><br>0.68<br>0.57-0.82	0.65<br>0.50-0.85	0.61<br>0.51, 0.72	74	17.7<br>6.2 - 71.6	
CANVAS [40] (Canaglifozin)	• T2DM<br>• ≥30yo+CVD or<br>• ≥50 yo+2 CVRF	10142 (6509/3633)	63	3.6	3 points MACE**	0.86<br>0.75–0.97<br>0.02<br>(for superiority)	0.87<br>0.72–1.06<br><br>0.87<br>0.74–1.01	0.67<br>0.52–0.87	0.60<br>0.47–0.77	77	12.4<br>6.71–40.9	
DECLARE [41] (Dapaglifozin)	• T2DM<br>• >40 years<br>• HbA1c >6.5% <12.0%<br>• eGFR ≥ 60 or multiple CVRF or High CV Risk*	17160 (10783/6422)	64	4.2	3 points MACE** and composite CVD or HFh	MACE<br>0.93<br>0.84−1.03<br>0,17<br><br>CVD or HFh<br>0.83<br>0.73−0.95<br>0,005	0.93<br>0.82−1.04<br><br>NA	0.73<br>0.61−0.88	0.53<br>0.43−0.66	85	13,1<br>6.0-43.6	
VERTIS [37] (Ertuglifozin)	• T2DM<br>• ≥40 yo, HbA1c<br>• 7.0 -10.5%,<br>• eGFR ≥30 ml, High CV Risk*	8246 (5769/477)	64	3.5	3 points MACE**	0.97<br>0.85–1.11<br><0.001	0.92<br>0.77–1.11<br><br>NA	0.70<br>0.54-0.90	0.81<br>0.63–1.04	75.9	1.31<br>1.23-1.38<br>mg/mmol<br>11,76<br>10,87-12,1<br>mg/g	
CREDENCE [42] (Canaglifozin)	• T2DM<br>• 30 years<br>• HbA1c 6.5 - 12.0% +<br>• eGFR ≥30 to <90 ml	4401 (2907/1494)	63	2.6 2	Composite renal adverse outcomes***	0.70<br>0.59–0.82<br>0.00001	0.78<br>0.61–1.00<br><br>0,83<br>0.68–1.02	0.61<br>0.47–0.80	0.73<br>0.61–0.87	56.2	9,27<br>463–18,33	

	(Prespecified ~60% inclusion of patients with eGFR 30 to <60 + UACR >300 to 5000.											
DAPA-CKD [43,44] (Dapaglifozin)	• Patients with or without T2DM<br>• eGFR ≥25 to 75 ml + UACR 200-5000	4304 (2879/1425)	63	2.4	Composite renal adverse outcomes[***]	0.61<br>0.51–0.72<br><0.001	0.81<br>0.58–1.12<br><br>0.69<br>0.53–0.88	NA<br>(Dapa 35 vs PCB 58)	0.56<br>0.45–0.68	43	9,65<br>4,72–19,03	18 months (curves started to diverge after 180 days)
DAPA-HF [28] (Dapaglifozin)	• With or without T2DM<br>• eGFR threshold ≥30 ml +<br>• LVEF ≤40%<br>• NYHA II-IV<br>• NT-proBNP ≥600 pg/ml (or ≥400 if hospitalized for HF in the previous year or ≥ 900 pg/ml if AF)	4744 (3635/1109)	66	1.5<br>1	Composite Hfh or CVD	0.74<br>0.65- 0.85<br><0.001	0.82<br>0.69 - 0.98<br><br>0.83<br>0.71 -0.97	0.70<br>0.59-0.83	NA<br>(DAPA 6,5% vs PCB 7,2%)	65.8	NA	28 days
EMPEROR reduced [29] (Empaglifozin)	• With or without T2DM<br>• eGFR ≥ 20 ml<br>• LVEF ≤30% and NT-proBNP ≥ 600 pg /ml or<br>• LVEF 31 - 35% and NT-proBNP ≥ 1000 pg/ml or<br>• LVEF 36 to 40% and NT-proBNP ≥2500<br>• In AF patients NT-proBNP thresholds doubled	3730 (2837/893)	67	1.4	Composite Hfh or CVD	0.75<br>0.65 -0.86<br><0.001	0.92<br>0.75 -1.12<br><br>0.92<br>0.77 -1.10	0.69<br>0.59-0.81	0.50<br>0.32 to 0.77	62	NA	12 days (stable after 34 days)
EMPEROR preserved [30] (Empaglifozin)	• LVEF>40%+<br>• eGFR ≥ 20	5988 (3312/2676)	72	2.0<br>1	Composite HFh or CVD	0.79<br>0.69–0.90<br><0.001	0.91<br>0.76–1.09	0.71<br>0.60–0.83	(renal endpoint based on>40% eGFR drop)<br>0.95	83	NA	18 days

	• NT-proBNP > 300 pg/ml (>900 if AF)						1.00<br>0.87–1.15		0.73–1.24<br><br>(renal endpoint based on>50% eGFR drop + renal death)<br>0.78<br>0.54-1.13			
SOLOIST WHF [35] (Sotaglifozin)	• T2DM<br>• HF Hospitalization needing intravenous diuretic therapy<br>• eGFR ≥30 ml	1222<br>(810/412)	69	0.7<br>7	Composite HFh or HF urgent visit or CVD	0,67<br>0.52–0.85<br><0.001	0.84<br>0.58-1.22<br><br>NA	0.67<br>0.55–0.82	NA	90.5	NA	
SCORED [36] (Sotaglifozin)	• T2DM<br>• CKD<br>• CVRF/High CV Risk*	10584<br>(7830/2754)	69	1.7	Composite HFh or HF urgent visit or CVD	0.74<br>0.63–0.88<br><0.001	0.90<br>0.73–1.12<br><br>0.99<br>0.83–1.18	0.64<br>0.49-0.83	0.71<br>0.46–1.08	90.5	7,4<br>1,8–4,86	
DAPA CKD [33] (Dapaglifozin)	• With or without T2DM<br>• GFR ≥25 to 75<br>• ACR 200 - 5000 (+HF subgroup)	468<br>(Female 36%)	65	2.4	Composite renal adverse outcomes***	0.58<br>0.37–0.91	NA<br>0.56<br>0.34–0.93	0.62<br>0.35–1.10	Primary EP	43.2	940<br>455.6-1,846.9	
DAPA CKD [33] (Dapaglifozin)	• With or without T2DM<br>• eGFR ≥25 to 75 ml<br>• ACR 200 to 5000 (No HF subgroup)	4304<br>(Female 32,7%)	61	2.4	Composite renal adverse outcomes***	0.62<br>0.51–0.75	NA<br>0.73<br>0.54–0.97	0.40<br>0.23–0.70	Primary EP	43.1	949.8<br>480.4-1,890	
